# The Role of Emergent Literacy Assessment in Brazilian Portuguese Literacy Acquisition during COVID-19

**DOI:** 10.3390/bs13060510

**Published:** 2023-06-19

**Authors:** Érica Prates Krás Borges, Gabriella Koltermann, Carla Alexandra da Silva Moita Minervino, Jerusa Fumagalli de Salles

**Affiliations:** 1 Institute of Psychology, Federal University of Rio Grande do Sul, Porto Alegre 90035-003, Brazil; 2 Psychopedagogy Department, Federal University of Paraíba, João Pessoa 58050-585, Brazil

**Keywords:** literacy acquisition, reading, spelling, emergent literacy, COVID-19

## Abstract

The contributions of emergent literacy skills to reading and writing development have been evidenced in different linguistic contexts. The worsening of the Brazil literacy scenario during the pandemic denoted the importance of a better understanding of these contributions’ specificities in Brazilian Portuguese to support evidence-based mitigation strategies. This study aimed to analyze the associations between emergent literacy components (emergent writing, alphabet knowledge, vocabulary, and phonological awareness) and word/pseudoword reading and spelling performance in first grade students during COVID-19. A total of 42 children (M_age_ = 6.29 years, SD = 0.45, 52.4% female) participated remotely in this study. Correlations and multilinear regression analyses were conducted. The results show significant associations between emergent literacy components and reading and spelling performance. Stronger associations were found with specific emergent skills such as letter writing, spontaneous writing, letter-sound production, and alliteration. Regression models indicated that children’s performance in early literacy skills explained 49% of the variance in reading and 55% of the variance in spelling. This study highlighted the role of emergent writing and alphabet knowledge as reading and spelling predictors during literacy acquisition in Brazilian Portuguese. Implications for educational context and directions for remediating the negative impact of the pandemic on learning were discussed.

## 1. Introduction

The predictive role of emergent literacy skills on future reading and writing performance has been evidenced in various linguistic contexts [1,2,3,4,5]. According to Lonigan [6], emergent literacy can be defined as skills, knowledge, and attitudes that children learn about reading and writing before they learn to read or write in the conventional sense. The assessment of emergent literacy skills contributes to the early identification of children at risk of developing reading and writing difficulties, supporting monitoring the students’ learning process, and highlighting effective teaching and intervention practices [1,5,6,7]. However, the type of emergent skill that is more strongly related to reading and writing performance varies according to the student’s grade, as education level is an important factor that influences the strength of associations between emergent literacy and written language [8,9]. Thus, in order to properly select emergent literacy tasks to assess children’s performance, it is imperative to be mindful of the schooling effect on emergent literacy assessment.

This effect can be partially explained by the bidirectional relation between emergent literacy skills and the literacy acquisition process. While the stimulation of emergent literacy skills during preschool may favor the reading and writing learning process in elementary school, the contact with classroom formal instruction during literacy acquisition may boost the development of emergent literacy skills [6,9,10,11]. For instance, when a child learns the alphabetic principle, their letter-sound knowledge and phonemic awareness skills also increase, just as the more proficient in reading and writing a child gets, the richer their vocabulary becomes [9,10,11,12].

Rohde’s Comprehensive Emergent Literacy Model [13] portrays emergent literacy in four interrelated components: phonological awareness, oral language, print awareness, and emergent writing. In the phonological awareness component, skills at syllabic and intra-syllabic levels are better reading and writing predictors during preschool, and as the student navigates through first grade, phonemic-level skills start to show stronger associations [10,14,15,16,17]. In the oral language component, contributions to reading and writing vary by skill type, and more complex skills, such as text-level auditory comprehension and narrative discourse, show stronger associations than simpler skills, such as receptive and expressive vocabulary [6,13,18]. Furthermore, associations between reading and oral language tend to be more consistent with reading comprehension than with word reading precision, and the higher the child’s grade, the stronger the associations between oral language and reading comprehension [19,20,21].

While the contributions of phonological awareness and oral language to reading and writing seem to prevail throughout school grades, the contributions of print awareness and emergent writing seem to decrease as the students reach more advanced school levels and improve their reading and writing proficiency [22,23,24,25,26]. However, differences in associations can be found when assessing different component skills, such as in alphabet knowledge, in which some studies suggest that the letter-sound production skill can be more strongly correlated to reading and writing than the letter naming skill [27,28]. In the emergent writing component, a meta-analysis conducted with English speaking children highlighted the specific contributions of name writing and letter writing to reading and writing performance [29].

Research conducted with Brazilian children has shown significant associations between phonological awareness and oral language and reading and writing performance during literacy acquisition [30,31,32,33]. On the other hand, print awareness and emergent writing have been less studied in Brazilian literature [7]. Understanding how each emergent literacy component contribute to written language development during literacy acquisition in Portuguese is relevant since associations strengths and significance levels may vary in different linguistics contexts [2,23,34,35]. In addition, the identification of which specific skills amid the different components of emergent literacy are more strongly correlated to reading and writing performance supports the development of evidence-based interventions and assessments adequate for the Brazilian context.

### 1.1. COVID-19 Impacts on Brazilian Literacy Scenario

In 2020, most educational settings were temporarily closed in an attempt to control the COVID-19 pandemic, which led to approximately 91% of students around the world being kept away from school [36]. In Brazil, school closure affected around 35.2 million children and adolescents, mostly public school students [37]. School closure can be considered particularly harmful to first grade children that are at the beginning of their literacy acquisition process as formal classroom instruction is essential to written language development [38,39,40].

According to the Organization for Economic Co-Operation and Development [41], countries that held the worst Program for International Student Assessment (PISA) reading proficiency scores in 2018 were the ones in which schools remained closed the longest in 2020. The report pointed out that reading performance explained 61% of the variance in school closure time, indicating that students that had the worst reading proficiency before the pandemic were the ones that had fewer learning opportunities during COVID-19 [41]. Among OECD members, Brazil was the country that held the longest school closure time, which was up to 25 weeks in 2020 [42].

Even though data on the pandemic effects on Brazilian students learning are still limited, one of the first studies carried out by the government of São Paulo (Brazil) predicted a delay in children reading learning by 2.46 years [43]. According to preliminary Brazilian large-scale assessment program (Sistema de Avaliação da Educação Básica—SAEB) results from 2021, the percentage of children that finished second grade without meeting any of the required reading and spelling skills tripled, rising from 5% to 15% [44]. In addition, the number of children that could only read words with visual aid and spell the first letters of words by the end of second grade went from 10% to 20% [44]. Thus, the preliminary results evidence significant negative impacts of the pandemic on Brazilian children’s reading and writing learning process.

### 1.2. The Present Study

The investment in evidence-based instruction and assessment practices in the context of reading and writing can be considered an essential measurement for mitigating the worsening of Brazil’s literacy status. As the benefits of both the early identification of children at risk of developing written language difficulties and the implementation of effective teaching strategies to help the prevention of said difficulties are recognized, the development of new studies to support these practices is necessary to enable their implementation [1,3,45].

Emergent literacy skills performance is considered an important indicator of the literacy acquisition process of first grade students in various linguistic contexts, including Portuguese [2,30,34]. However, there is no consensus regarding the specific contributions of the emergent literacy components to reading and writing performance in Brazilian Portuguese, as well as of the different skills that comprise each component. Understanding how these relationships occur in the Brazilian context is relevant as it allows for the development of teaching and monitoring practices that are appropriate for the students’ linguistic environment.

The present study had the main goal of analyzing the relation between first graders’ performance in emergent literacy components (emergent writing, alphabet knowledge, vocabulary, and phonological awareness) and their word and pseudoword reading and spelling performance. The first specific goal of the study was to describe the children’s performance in administered tasks (mean score and percentile ranges) to characterize the students skills’ profile. The second specific goal was to analyze differences in the associations between emergent literacy and written language by emergent skill type. The third specific goal was to investigate the predictive role of emergent literacy skills in students’ reading and spelling performance in order to highlight the cognitive–linguistic skills that contribute the most to the early identification and prevention of reading and writing difficulties in Portuguese.

Some hypotheses were raised regarding the expected associations between the variables of interest. First, as Portuguese can be considered an orthography of intermediate transparency, a prevalent use of phoneme–grapheme conversion strategies (phonological route) in reading and writing is expected, with fewer uses of lexical strategies (lexical route) at the beginning of literacy acquisition [23,46]. Thus, it can be hypothesized that the emergent literacy components that are more related to phonological processing skills and/or that contribute the most to the phoneme–grapheme conversion process should present stronger associations with reading and writing performance in first grade students. Therefore, components such as emergent writing, alphabet knowledge, and phonological awareness are expected to be more strongly related to reading and writing than the vocabulary component [6,34].

Regarding the specific emergent skills analysis, some differences within associations with reading and writing were hypothesized considering the effect of the education level of the assessed students (first grade, i.e., the beginning of the literacy acquisition process) and type of orthography (intermediate transparency): (a) letter-sound knowledge should be more strongly associated than letter-name knowledge [27,28]; (b) phoneme awareness (alliteration) will show stronger associations with reading and writing than syllabic awareness (syllabic manipulation) [14,15,16]; (c) letter writing and spontaneous writing will be more strongly associated than name writing [24,47]. Additionally, it is expected that the emergent literacy components, specifically the performance in the previously highlighted skills, will be significant predictors of students’ reading and writing outcomes [6,30,32].

## 2. Materials and Methods

This cross-sectional study was conducted remotely between the months of May 2021 and October 2021, during the COVID-19 pandemic. Data collection procedures were managed by two research centers located in the states of Rio Grande do Sul and Paraíba (Brazil).

### 2.1. Participants

Convenience sampling (social media invites and contact with local schools) was implemented to recruit participants. Inclusion criteria were adopted as follows: (a) being a first-grade student; (b) absence of uncorrected sight and/or hearing impairments; (c) absence of psychological, psychiatric, and/or neurological diagnosis as reported by the parents. Families that had access to a computer with camera, microphone, and Internet were considered eligible for this study. Availability to accompany the child during a remote assessment session through video conference was considered an eligibility criterion as well. Of the 76 families that volunteered to participate in the study, 13 did not complete data collection procedures and 21 were excluded for data analysis due to not meeting inclusion or eligibility criteria.

Final sample consisted of 42 first grade students (M_age_ = 6.29, SD = 0.45, Range = 6–7 years old, 52.4% female), mostly enrolled in private schools (83.3%). During school closure, 90.5% of participants attended remote classes, of which 80.6% attended daily and 19.4% attended twice a week. Regarding family context, the majority of caregivers had a complete higher education degree (66.7%) and came from an upper middle class socioeconomic background (57.1% from B1 and B2 classification) according to the Brazilian Criterion for Economic Classification [48].

### 2.2. Instruments and Questionnaires

Due to the social distancing measures adopted during the COVID-19 pandemic, all instruments and questionnaires were administered remotely. Parent reports consisted of the Child’s Socioeconomic, Health and Education Conditions Questionnaire and the Brazilian Criterion for Economic Classification [48], which were administered online through the Google Forms platform. The instruments used to assess children’s performance in emerging literacy skills, reading, and spelling went through an adaptation process for remote administration.

#### 2.2.1. Early Literacy Skills Assessment Tool

The Early Literacy Skills Assessment Tool [49] evaluates five emergent literacy components: alphabet knowledge, emergent reading, emergent writing, phonological awareness, and vocabulary. The instrument has preliminary validity for preschool students in the face-to-face version [50]. The components and specific tasks adapted for the study were: (a) emergent writing: name writing, letter writing, and spontaneous writing; (b) alphabet knowledge: letter-name identification, letter-sound identification, letter naming, and letter-sound production; (c) vocabulary: picture naming; (d) phonological awareness: alliteration identification and syllabic manipulation (addition and subtraction).

The instrument was adapted for remote assessment with the transposition of stimuli to PowerPoint. In the emergent writing tasks, the participants were advised to write their answers on paper. In the spontaneous writing task, the child was given three minutes to write as many words as they knew. Each correct word scored one point, and wrong words, repeated words, and/or derivatives of the same word scored zero points. The raw scores were standardized based on the sample percentiles analysis (1 point = 1 to 2 correct words; 2 points = 3 to 4; 3 points = 4 to 6; 4 points = 7 to 9; and 5 points = 10 or more).

#### 2.2.2. Word/Pseudoword Reading Task

The Word/Pseudoword Reading Task [51] evaluates reading performance in children from six to twelve years old. The task consists of thirty-nine words and twenty pseudowords controlled by regularity, length, frequency, and lexicality. The face-to face version of the instrument has validity and reliability evidence, as well as standardized norms by age and school level.

The remote version of the reading task had twenty words and ten pseudowords to optimize the application time. The criteria used to exclude stimuli was the difficulty level of the words for first graders. Low-frequency words and lengthier pseudowords were excluded considering the incidence of frequency and length effects in this schooling range [52,53]. For remote administration, words and pseudowords were presented individually in PowerPoint (font Arial, size 40, capital letters). The screen sharing tool was used to present the test and the child was asked to read aloud the stimulus projected on the screen. The real words were presented in four blocks: short regulars, short irregulars, long regulars, and long irregulars. The pseudowords were presented in a single block. After reading each block, the participant indicated whether they would like to continue or interrupt the task.

#### 2.2.3. Word/Pseudoword Spelling Task

The Word/Pseudoword Writing Task from the Children’s Brief Neuropsychological Assessment Battery [54] evaluates spelling performance in children from six to twelve years old. The task consists of fourteen words and five pseudowords controlled by regularity, length, frequency, and lexicality. The face-to face version of the instrument has validity and reliability evidence, as well as standardized norms by age. In the remote version, the researcher dictated the words through videoconference and the participant wrote their answers on paper.

### 2.3. General Procedures

#### 2.3.1. Pilot Study

A pilot study was conducted to test the remote adaptation of the instruments during April 2021. Five first grade children responded to the remote version of the Early Literacy Skills Assessment Tool and the Word/Pseudoword Reading and Writing tasks. Results from the pilot study indicated the need to modify specific tasks in the Early Literacy Skills Assessment Tool. In the alphabet knowledge and phonological awareness tasks, red flags were added to indicate the target stimuli, as the researcher could not point to the target stimuli in a video conference setting. The vocabulary task was reduced from 96 to 48 stimuli to optimize administration time. There was no need to modify the other tasks and instruments as the video conference setting had little to no intervention in their administration procedures.

#### 2.3.2. Data Collection Procedures

Data collection took place during the months of May 2021 and October 2021. During this period, social distancing measures were still implemented in educational context, and elementary schools were operating with remote and/or hybrid classes. Families that were interested in participating in the study were asked to sign a consent form and to fill out the parent reports. Remote evaluation sessions were scheduled individually, and parents were given specific instructions to make sure that the child was in a quiet room with access to a computer, pencil, and paper. During the assessment, both the child’s camera and microphone were asked to stay turned on, and an adult had to be available to assist the child, if necessary, without intervening with their answers.

The remote evaluation sessions were conducted by two psychologists specialized in child neuropsychological assessment from the Rio Grande do Sul research center and five psychology students from the Paraíba research center that received proper training to administer the tasks. The Google Meet platform was used to administer the instruments, and each individual session had an average duration of 50 min. To standardize the collection procedures, all sessions were conducted using a computer, and the use of cell phones or tablets was not allowed. The screen sharing tool was used to present the stimuli to the participants. The researcher scored the children’s answers during the evaluation as the remote session was not recorded. Instrument administration order was: Early Literacy Skills Assessment Tool, Reading task, and Writing task.

After the remote session, parents were asked to send a picture to the research team of the child’s emergent writing and spelling under dictation answers. Families that completed their participation in the study received informational booklets on how to encourage literacy and numeracy development in the family environment as well as individual reports on their child’s performance in the administered tasks.

#### 2.3.3. Data Analysis Procedures

The software SPSS 26 was used for data analysis. Preliminary analysis was conducted to verify data homogeneity and to identify discrepancies in intragroup performance. Due to the non-parametric data distribution, risk of bias in the sample’s performance by type of school (public and private), socioeconomic level, and month of collection was assessed using the Mann–Whitney test.

In view of the fact that the instruments used to assess reading and writing performance were adapted to remote administration and that the equivalence of remote and face-to-face versions was not yet verified, it was opted not to use normative data to interpret participants performance in this study. Likewise, the emergent literacy test did not have normative data for first grade students. Therefore, the sample’s performance profile was presented through descriptive analysis, score distribution by percentile range, and the percentage of children who were allocated to each percentile.

To analyze the associations between emergent literacy components, and reading and writing performances, Kendall’s tau-b correlation was performed. Emergent literacy variables were analyzed by components and by skill type. In the emergent writing component, name writing was a dichotomous variable, so to assess its association with other variables, point-biserial correlation coefficient was used. The alphabet knowledge component was divided into two separate variables: (a) letter-name knowledge, including letter-name identification and letter naming skills, and (b) letter-sound knowledge, including letter-sound identification and letter-sound production skills. Correlations’ effect sizes were interpreted according to Ambiel et al.’s [55] proposed values, in which 0.1 = weak, 0.4 = moderate, 0.7 = strong, 0.8 = very strong. Fisher’s r-to-z test was used to analyze differences among correlations coefficients [56].

After the correlation analysis, variables that were significantly and moderately correlated were included in the regression models. Multilinear regression analyses were conducted with the forward method to assess the predictive power of emergent literacy skills in reading and spelling performance. The Durbin–Watson coefficient was used to analyze the independence of residues and the Cook and Mahalanobis distances were used to assess the outliers’ effect. Finally, multicollinearity between predictors was measured using the variance inflation factor (VIF).

### 2.4. Ethical Considerations

The Ethics Committee of the Federal University of Rio Grande do Sul approved this study (CAAE 42544621.1.0000.5334). Participation in the study was voluntary through the parents’ signing of the free and informed consent form and the children’s oral acceptance of the free and informed assent form. Participants’ materials and protocols were safely stored in a private online account with restricted access. Parents of children that performed poorly in the administered tasks were contacted and the participant was referred to a specialist for a complete neuropsychological assessment. When cognitive–linguistic alterations were identified, the participant was referred to specialized intervention.

## 3. Results

Preliminary analysis showed that most variables had no significant differences in intragroup performance by school type, socioeconomic status, and month of data collection, indicating a low risk of bias. Performance by school type differed only for letter-sound knowledge (U = 62.000, z = −2.05, *p* = 004, r = 0.31) and vocabulary (U = 29.500, z = −2.89, *p* = 0.007, r = 0.46), with higher scores in private school children. Children from a higher socioeconomic status also presented higher scores in letter-sound knowledge tasks (U = 23.500, z = −3.21, *p* = 0.001, r = 0.6). Considering the preliminary results and the reduced sample size, we opted not to divide participants by school type since the pandemic context made it infeasible to expand the sample size.

### 3.1. Sample’s Performance in Administered Tasks

Table 1 presents the sample’s performance in emergent literacy components. In the emergent writing component, a ceiling effect was observed in the name writing task, with 90% of the participants writing their name correctly. In the letter writing task, there was a high variability in the sample’s performance as a similar number of participants were placed at the extreme points of the score distribution. The spontaneous writing task proved to be more difficult for children in relation to other emergent writing tasks as approximately 15% of participants could not write any correct words.

In the alphabet knowledge component, which was divided into letter-name and letter-sound knowledge, a ceiling effect was observed in the letter-name and letter-sound identification tasks. Children had more difficulties with the letter-sound production task than the letter naming task, as noted by the score distribution by percentile (lower scores and higher standard deviations). While 40% of first grade children were able to name all the 26 alphabet letters, only 25% were able to produce their corresponding sound. Participants’ performance in vocabulary and phonological awareness tasks also indicated a ceiling effect in some cases. However, 10–15% of children got only half of the phonological awareness items right, suggesting that despite the ceiling effect observed in more than half of the cases, some children showed a poor performance.

Table 2 presents the sample’s performance in reading and spelling tasks. Regarding the word reading task, approximately 30% of the children were only able to read at least seven words, and approximately 15% of children could only read two or fewer words. Considering that the task consisted of twenty words in total, these results suggest that approximately half of the sample could not read most of the presented words. The children’s pseudoword reading performance was similar to their word reading performance, with a similar proportion of participants who performed poorly. A lower performance in the spelling task was noted in relation to the reading task, and many children (approximately 40%) could not write any of the five pseudowords correctly. Additionally, around 20% of participants were only able to write two or fewer words under dictation from a total of fourteen words.

### 3.2. Associations between Emergent Literacy Skills and Reading and Spelling

Table 3 presents the correlations between emergent literacy skills and word/pseudoword reading and spelling. The emergent writing and letter-sound knowledge components were significantly and moderately correlated to word/pseudoword reading and writing performance and were the emergent literacy components that were the most strongly related to written language. Significant associations were also found between the letter-name knowledge component and reading and writing performance. On the other hand, vocabulary and phonological awareness skills had fewer significant associations with reading and writing performance. Vocabulary performance was weakly correlated to word reading and pseudoword writing, whereas phonological awareness was only significantly associated with reading skills.

In the emergent writing component, association analysis by skill type indicated that letter writing and spontaneous writing were moderately associated with written language performance, and the Fisher’s r-to-z test did not find any significant differences between the correlation’s coefficients. Meanwhile, the name writing skill was only associated with other emergent literacy skills, such as letter writing, letter-sound identification, and phonological awareness. In the letter-sound component, the letter-sound identification skill was not significantly associated with reading and writing performance, as there was a ceiling effect in this task. In view of this, in this component, only the letter-sound production skill was significantly associated with written language.

As for the letter-name knowledge component, it was observed that both letter-name identification and letter naming skills were significantly, although weakly, associated with reading and writing performance (except for the association between letter-name identification and pseudowords writing, which was not significant). Just as in the emergent writing component, there were no significant differences in correlation’s coefficients between letter-name knowledge skills. Finally, in the phonological awareness component, only the alliteration skill was significantly correlated with reading and writing, presenting weak correlations with reading performance and moderate correlations with writing performance.

### 3.3. Contributions of Emergent Literacy for Reading and Spelling Performance

Based on the results of the correlation analyses, multiple linear regressions were performed to investigate the extent to which the performance in emergent literacy skills contributes to the performance in word/pseudoword reading and writing. In the reading model, performances in emergent writing (combined score in letter writing and spontaneous writing tasks) and letter-sound production were included and, in the writing model, in addition to these two predictors, the alliteration skill was also added. Reading and writing performances were analyzed using a combined score for words and pseudowords due to the small number of items in each category as it limited a more specific analysis of the emergent skills contributions to word and pseudoword processing.

The results show that emergent writing and letter-sound knowledge were significant predictors of reading (F(2.29) = 16.04, *p* < 0.001, R^2^ adjusted= 0.49) and spelling outcomes (F(2.36) = 24.202, *p* < 0.001, R^2^ adjusted= 0.55). More specifically, the analysis shows that children’s performance in said emergent literacy skills explained 49% of the variance in word/pseudoword reading performance and 55% of the variance in word/pseudoword writing performance. In the spelling model, the alliteration skill had no significant contributions and was excluded from the model (B = 0.177, t = 1.416, *p* = 0.166). Table 4 and Table 5 present the coefficient to every significant predictor. Outliers’ analysis and residuals’ independence analysis indicated no unfavorable conditions for the regression models. Multicollinearity analysis evidenced a variance inflation factor (VIF) of 1.45 for the reading model and 1.65 for the spelling model, suggesting that the association between the predictors are not strong enough to impact the regression models.

## 4. Discussion

The present study aimed to analyze the associations between first graders’ performance in the emergent literacy components and their word/pseudoword reading and writing performances. The first specific aim was to describe the students’ performance in administered tasks to characterize their skills’ profile. The study also explored the possible differences in associations between emergent literacy and written language by skill type within the emergent literacy components, as well as analyzed the predictive role of emergent literacy skills in word/pseudoword reading and writing performance.

### 4.1. First Grade Students Skills’ Profile during COVID-19

The descriptive analysis of the students’ skills’ profile indicated that a considerable number of children had a low performance in reading and spelling tasks, with approximately 15% of the participants being allocated to the lowest percentiles. A low performance was also observed in the emergent literacy assessment, in which approximately a quarter of the children had difficulty writing the letters of the alphabet and approximately 15% of the children were unable to write any words spontaneously. These results become more alarming when we consider that the sample was composed mostly of upper middle class private school students and that the adapted reading task was composed only of real words with higher-frequency and short pseudowords.

Although assessing the pandemic’s impact on learning was not a specific goal of this study, a great parcel of the sample showed a poor performance in skills that should be mastered by first graders according to the national literacy benchmarks [57]. In this context, the children skill’s profile presented in this study illustrates the gap in the Brazilian literacy scenario and emphasizes the importance of evidence-based instruction and assessment to mitigate the pandemic’s impact on learning [1,3,45].

### 4.2. Associations between Emergent Literacy Skills and Reading and Spelling

The results evidence significant associations between most emergent literacy skills and children’s performance in words/pseudoword reading and writing. The emergent writing and letter-sound knowledge components showed the strongest associations in the study, followed by letter-name knowledge, phonological awareness, and vocabulary.

In the emergent writing component, only the name writing skill was not significantly associated with reading and spelling performance. In this task, most participants could write their own name correctly. As the frequency in which a child reads, listens, and practices their own name writing tends to be a lot higher than other words, a large number of children master the name writing skill during preschool, before the beginning of the formal literacy acquisition process [24,47]. While the name writing skill was only associated with other emergent literacy skills (letter writing, letter-sound identification, and phonological awareness), letter writing and spontaneous writing were moderately associated with reading and spelling performance. These findings suggest that, even though name writing is not directly and consistently associated with written language in first grades, assessing this skill can be a way to quickly screen children’s performance in emerging literacy, as it is indeed associated with alphabet knowledge and phonological awareness [24,47].

Furthermore, the results emphasize the moderate strength of the associations between emergent writing skills (letter writing and spontaneous writing) and student’s reading and spelling performance during the literacy acquisition process in Portuguese. Considering that emergent writing accounts for a lower amount of research in the emergent literacy field and that most studies conducted with Portuguese speaking children only assess the invented spelling skill, these findings evidence the contributions of letter writing and spontaneous writing to the literacy acquisition process in a Brazilian context [7,47].

In the alphabet knowledge component, which was divided into letter-name knowledge and letter-sound knowledge, stronger associations were found with letter-sound production than with letter naming. Even though there is no consensus regarding possible differences in the strength of these skills’ contributions, some studies suggest that the ability to produce the sound of letters is more relevant to reading and spelling performance than the ability to name letters, considering it has a closer relationship with the alphabetic principle’s learning process [27,28].

Even so, it is possible that the strength of the associations between alphabet knowledge and written language is influenced by the child’s linguistic context and by the way in which the emergent skills are assessed [26,34]. In more transparent languages, a ceiling effect can be more quickly observed in alphabet knowledge performance if the task does not have an adequate difficulty level [26]. This effect was found in the letter-name and letter-sound identification tasks, suggesting that these tasks may not be appropriate to assess first grade children. As the instrument used to assess emergent literacy was originally made for preschoolers, the selected items were possibly not sensible for assessing the first grade students’ performance.

In the phonological awareness component, only the alliteration skill was associated with reading and spelling performance, whereas the syllabic addition and subtraction skills did not show significant associations. It is possible that the type of task was not adequate for assessing this component in first graders as syllabic awareness skills are usually acquired before the beginning of the literacy process [16]. Considering the effect of education level on phonological awareness assessment, different studies indicate that, after the beginning of formal instruction in reading and writing, skills at the phoneme level begin to show stronger associations with written language than skills at the syllable level [10,14,15,16,17]. Since the only skill that was related to written language in this study was the ability to identify the initial phoneme of a word (alliteration), the findings reinforce the importance of prioritizing the assessment of phonemic awareness in first grade students for a more adequate evaluation.

Although the phonological awareness component as assessed in this study has shown weaker and inconsistent associations than the emergent spelling and alphabet knowledge components, the role of phonological awareness in written language development is widely recognized in both international and national literature [6,30,32,33,58]. Therefore, we understand that this study’s divergent results may reflect the limitations in which this component was evaluated, considering how the phonological awareness tasks were not fit for a first-grade sample.

In this study, an expressive vocabulary task was used as a measure of oral language. The vocabulary component had the least amount of significant correlations with reading and spelling performance, being only weakly associated with word reading and pseudoword writing. While some evidence in the Brazilian context indicates significant associations between expressive vocabulary measured during preschool and word reading assessed in the first grade [30,32], there is no consensus in the literature regarding the relationship between vocabulary and written language after the beginning of the literacy acquisition process. Some studies point to a schooling effect and a measure effect, indicating that, during preschool, there are stronger associations between vocabulary and word decoding and that, as the school level increases, the relationship with decoding weakens and vocabulary becomes more strongly associated with reading comprehension [19,20,21]. Furthermore, it is important to consider the ceiling effect found in some cases in the vocabulary assessment, which may also have influenced the strength of associations.

In this context, it is possible that the weak association between vocabulary and reading and spelling performance is a consequence of the previously mentioned effects and that this skill is possibly not as important for decoding in the first grade as other skills evaluated in the study. Granted that the results on vocabulary performance presented in this study contribute to expanding the knowledge about the relationship between oral language and written language during the literacy acquisition process, they are not exhaustive. Some studies suggest that more complex oral language skills may have stronger associations with written language than simpler measures such as expressive vocabulary tasks, indicating the need for further research for a more comprehensive understanding of the contributions from this emerging literacy component to reading and spelling performance [6,13,18].

### 4.3. Contributions of Emergent Literacy to Written Language and Implications for the Literacy Scenario

Based on the association analyses, the predictive role of emergent literacy skills on reading and spelling outcomes was verified. The regression models indicated significant contributions of emergent writing and letter-sound production to the reading and spelling performance of first grade students. The performance in emergent literacy skills explained 49% of the variance in reading and 55% of the variance in spelling, highlighting the predictive role of such skills regarding written language outcomes.

As studies conducted in different cultural backgrounds state the role of emergent writing and alphabet knowledge as predictors of written language [7,29,59,60], findings from this study evidence that those skills are also significant predictors of reading and spelling performance in Brazilian Portuguese. As most studies conducted in Portuguese analyze the longitudinal impact of emergent literacy performance assessed during preschool [7,17,30,61,62,63], this study’s cross-sectional design allowed for a better understanding of emergent writing and alphabet knowledge contributions to reading and spelling performance assessed during the literacy acquisition process.

Facing the post-pandemic educational context, evidencing emergent literacy contributions to reading and writing supports the development of evidence-based assessment and instruction methods to help mitigate the gap in the literacy scenario [3,5,64]. Based on the study’s results, some educational practices are proposed:

1. Emergent writing and alphabet knowledge assessment can be implemented through quick and simple tasks and provide important information regarding the identification of children at risk for difficulties in their reading and writing learning process [65,66,67]. These tasks can be easily administered simultaneously in a classroom setting and can help teachers to identify the students’ learning gaps.
2. As predictors of reading and writing, the stimulation of emergent writing and alphabet knowledge during preschool can be seen as a preventive practice regarding difficulties in the literacy acquisition process, and teachers can also instruct caregivers on how to stimulate said skills at home [4,68,69,70].
3. In view of the contributions of letter-sound knowledge for reading and writing development, the study’s results emphasize the importance of systematic phonics instruction as a more effective method for teaching reading and writing, which can facilitate the student’s understanding of the alphabetic principle [71,72,73].

### 4.4. Limitations and Directions for Future Research

The reduced sample size prevented the execution of more robust analyses on the contributions of emergent literacy to written language. The analysis of the sample’s sociodemographic characteristics indicated that most of the assessed children were private school students that had a family background of high levels of maternal education and a high socioeconomic status. Hence, it is imperative to take into consideration that data from this study reflect a specific parcel of children and may not be representative of other contexts. Additionally, it is necessary to conduct new studies that include children from different socioeconomic levels and types of school for a better understanding of the environment’s influence on the relationship between emergent literacy and written language. The inclusion of contextual variables in explanatory models is relevant considering that some evidence points to the protective role of family literacy practices that encourage the development of emergent literacy for children at risk of reading and writing difficulties [64,70,74].

The use of remote versions of tasks originally developed for face-to-face administration without testing their equivalence limited the interpretation of the sample’s performance to a descriptive analysis and score distribution by percentile range. Thus, new studies should analyze the equivalence of the versions to verify the adequacy of the standardized norms developed before the COVID-19 pandemic, allowing for a more accurate interpretation of children’s performance in relation to what would be expected for their age and education range. In addition, the difficulty level of some emergent literacy tasks (phonological awareness, vocabulary, and letter-name and sound identification) proved to be inadequate for the sample’s education level, leading to a ceiling effect for part of the children. Thus, the tasks’ stimuli should be revised to optimize the use of the Early Literacy Skills Assessment Tool with first grade children.

Considering the ceiling effect on phonological awareness and vocabulary tasks, it was not possible to accurately analyze their associations with reading and writing performance. Therefore, new studies that seek to evaluate the contributions of the phonological awareness and oral language components to written language should use tasks appropriate to the sample’s education level, considering the schooling effect on emergent literacy performance [6,10]. More specifically, the literature points out that phonemic awareness, oral comprehension, or narrative expression at the discourse level would present stronger associations with written language in children after the beginning of the literacy acquisition process [6,14,16,18].

## 5. Conclusions

The present study evidenced significant associations between the emergent literacy components (emergent writing, alphabet knowledge, vocabulary, and phonological awareness) and the word/pseudoword reading and writing performance of first grade children. This study contributed to broadening the understanding of the relationship between emergent literacy and written language in Portuguese since it encompassed the assessment of all four components of the Comprehensive Emergent Literacy Model [13], in addition to detailing the associations for specific skills from each component. Regression models showed significant contributions of emergent writing and letter-sound production to reading and writing outcomes, and the children’s performance in emergent literacy skills explained approximately half of the variance in written language. Based on the results, its implications for the educational context were discussed and some educational practices were proposed for remediating the pandemic’s negative impact on the literacy scenario.

As the importance of evidence-based assessment is recognized, the accessibility to instruments with good psychometric properties suitable for the Brazilian context is essential for monitoring students and for accurately identifying children at risk for learning difficulties. Considering the suggestions for optimizing the Early Literacy Skills Assessment Tool for first grade children, we highlight the benefits of using the instrument as a standardized measure that allows for the assessment of different components of emerging literacy. Finally, the study evidenced the feasibility of remote reading and writing assessment in children and provided important data when describing first grade students’ performance in emergent literacy and written language during the pandemic.

## Figures and Tables

**Table 1:** Sample’s performance in emergent literacy tasks and score distribution by percentile.

Task	MS *	M (SD)	Median	10th		25th		50th		75th		90th	
				Score	%	Score	%	Score	%	Score	%	Score	%
Emergent Writing	32	23.39 (8.39)	27	8	14.6	20	24.3	27	24.3	30	12.2	31	14.6
Name Writing	1	0.90 (0.30)	1	0	9.8	1	-	1	-	1	-	1	90.2
Letter Writing	26	20.12 (7.33)	23	6	19.5	17	19.5	23	12.2	25	19.5	26	24.3
Spontaneous Writing	5	2.37 (1.51)	2	0	14.6	1	12.2	2	29.2	3	34.1	5	9.7
Letter-Name Knowledge	29	26.42 (4.01)	28	19	11.9	26	26.1	28	11.9	29	-	29	40.4
Letter-Name Identification	3	2.95 (0.21)	3	3	-	3	-	3	-	3	-	3	95.2
Letter Naming	26	23.48 (3.90)	25	17	11.9	23	26.1	25	11.9	26	-	26	40.4
Letter-Sound Knowledge	25	18.00 (6.68)	19.5	7	16.6	14	21.4	19	21.4	24	9.5	25	23.8
Letter-Sound Identification	3	2.79 (0.56)	3	2	7.1	3	-	3	-	3	-	3	85.7
Letter-Sound Production	22	15.21 (6.50)	16.5	5	14.2	11	23.8	16	28.5	22	-	22	26.1
Vocabulary	12	10.20 (1.52)	10	8	10	9	15	10	27.5	11	22.5	12	22.5
Phonological Awareness	4	3.24 (0.79)	3	2	14.6	3	-	3	39	4	-	4	43.9
Alliteration	4	3.54 (0.67)	4	2	9.8	3	26.8	4	-	4	-	4	63.4
Syllabic Addition	4	3.37 (0.79)	4	2	12.2	3	31.7	4	-	4	-	4	53.7
Syllabic Subtraction	12	10.20 (1.52)	10	8	10	9	15	10	27.5	11	22.5	12	22.5

* Maximum score.

**Table 2:** Sample’s performance in reading and spelling tasks and score distribution by percentile.

Task	MS *	M (SD)	Median	10th		25th		50th		75th		90th	
				Score	%	Score	%	Score	%	Score	%	Score	%
Reading—Total	30	15.61 (9.86)	13	2	9.1	5	30.3	13	27.2	25	15.1	28	12.1
Word Reading	20	9.50 (6.57)	8	0	17.6	3	29.4	8	20.5	15	14.7	18	17.6
Pseudoword Reading	10	5.68 (3.46)	6	0	13.5	2	29.7	6	24.3	9	16.2	10	16.2
Spelling—Total	19	7.59 (5.04)	8	0	17.1	3	31.7	8	17.1	12	4.8	13	21.9
Word Spelling	14	5.66 (3.62)	6	0	19.5	3	24.3	6	29.2	9	17.1	10	9.7
Pseudoword Spelling	5	1.93 (1.64)	2	0	-	0	41.4	2	12.2	3	29.2	4	17.1

* Maximum score.

**Table 3:** Correlations between emergent literacy skills and reading and spelling.

	1	2	3	4	5	6	7	8	9	10	11	12	13	14	15	16	17	18	19	20	21
1	-																				
2	0.95 **	-																			
3	0.86 **	0.79 **																			
4	0.64 **	0.65 **	0.71 **	-																	
5	0.63 **	0.64 **	0.68 **	0.91 **	-																
6	0.58 **	0.58 **	0.64 **	0.80 **	0.68 **	-															
7	0.49 **	0.53 **	0.52 **	0.57 **	0.57 **	0.51 **	-														
8	−0.00	0.12	−0.02	0.18	0.17	0.18	0.41 **	-													
9	0.47 **	0.52 **	0.50 **	0.55 **	0.53 **	0.50 **	0.85 **	0.36 *	-												
10	0.40 **	0.43 **	0.45 **	0.54 **	0.55 **	0.47 **	0.64 **	0.30	0.45 **	-											
11	0.36 **	0.36 **	0.31 *	0.38 **	0.39 **	0.31 **	0.45 **	0.12	0.51 **	0.21	-										
12	0.33 *	0.31 *	0.30 *	0.28 *	0.29 *	0.24	0.28 *	0.30	0.28 *	0.30 *	0.30 *	-									
13	0.36 **	0.36 **	0.31 *	0.38 **	0.39 **	0.31 **	0.45 **	0.10	0.51 **	0.21	1.00 **	0.30 *	-								
14	0.47 **	0.52 **	0.41 **	0.49 **	0.50 **	0.43 **	0.50 **	0.29	0.48 **	0.50 **	0.40 **	0.26 *	0.40 **	-							
15	−0.03	−0.01	−0.09	0.21	0.21	0.17	0.22	0.30 *	0.22	0.24	0.29 *	0.20	0.29 *	0.29 *	-						
16	0.49 **	0.53 **	0.44 **	0.49 **	0.49 **	0.42 **	0.49 **	0.27	0.47 **	0.51 **	0.37 **	0.26 *	0.37 **	0.97 **	0.22	-					
17	0.26	0.31 *	0.21	0.22	0.21	0.26 *	0.16	−0.07	0.22	0.05	0.18	0.42	0.18	0.29 *	−0.09	0.29 *	-				
18	0.16	0.17	0.20	0.29 *	0.30 *	0.34 **	0.24 *	0.31 *	0.28 *	0.31 *	0.05	0.14	0.05	0.27 *	0.24	0.28 *	0.29 *	-			
19	0.33 *	0.37 **	0.37 **	0.44 **	0.40 **	0.55 **	0.34 **	0.30	0.30 *	0.32 *	0.09	0.25	0.09	0.30 *	0.15	0.30 *	0.13	0.57 **	-		
20	0.03	−0.00	0.02	0.05	0.05	0.07	0.05	0.26	0.02	0.09	−0.04	0.12	−0.04	0.14	0.37 *	0.13	0.15	0.55 **	0.12	-	
21	0.09	0.09	0.06	0.16	0.20	0.12	0.23	0.04	0.28 *	0.21	0.08	0.25	0.08	0.21	0.32 *	0.21	0.216	0.55 **	0.14	0.24	-

* *p* < 0.05; ** *p* < 0.01; 1 = Word/pseudoword reading; 2 = Word reading; 3 = Pseudoword reading; 4 = Word/pseudoword spelling; 5 = Word spelling; 6 = Pseudoword spelling; 7 = Emergent writing; 8 = Name writing; 9 = Letter writing; 10 = Spontaneous word writing; 11 = Letter-name knowledge; 12 = Letter-name identification; 13 = Letter naming; 14 = Letter-sound knowledge; 15 = Letter-sound identification; 16 = Letter-sound production; 17 = Vocabulary; 18 = Phonological awareness; 19 = Alliteration; 20 = Syllabic addition; 21 = Syllabic subtraction.

**Table 4:** Word/pseudoword reading regression model coefficients.

	Unstandardized Coefficients		Standardized Coefficients				
Predictors	*B*	SE	B	t	*p*	R^2^	ΔR^2^
Constant	−7.691	4.366	-	−1.761	0.089	-	-
Emergent Writing	0.574	0.197	0.447	2.910	0.007	0.409	-
Letter-Sound Knowledge	0.584	0.239	0.375	2.439	0.021	0.492	0.083

**Table 5:** Word/pseudoword writing regression model coefficients.

	Unstandardized Coefficients		Standardized Coefficients				
Predictors	B	SE	B	t	*p*	R^2^	ΔR^2^
Constant	−5.097	1.901	-	−2.682	0.011	-	-
Emergent Writing	0.253	0.082	0.426	3.078	0.004	0.452	-
Letter-Sound Knowledge	0.351	0.116	0.416	3.011	0.005	0.550	0.098

## Data Availability

The data presented in this study are available on request from the corresponding author. The data are not publicly available as the Early Literacy Skills Assessment Tool was in the process of normative data development and publishing.
